# Supplementary material for: Attempts to Create Products with Increased Health-Promoting Potential Starting with Pinot Noir Pomace: Investigations on the Process and Its Methods
Source: Foods. 2022 Jul 6;11(14):1999. doi: 10.3390/foods11141999 (PMC9323902; doi:10.3390/foods11141999)
Supplement: Supplementary file 1 [file foods-11-01999-s001.zip › foods-1757154-supplementary.pdf]

Attempts to Create Products with Increased Health-Promoting Potential Starting with Pinot Noir Pomace: Investigations on the Process and Its Methods

Stephen Lo [1*], Lisa I. Pilkington [1], David Barker [1,2] and Bruno Fedrizzi [1,2,*]

1. School of Chemical Sciences, University of Auckland, 23 Symonds St, Auckland 1010, New Zealand; lisa.pilkington@auckland.ac.nz (L.I.P.); d.barker@auckland.ac.nz (D.B.); b.fedrizzi@auckland.ac.nz (B.F.)

2. Centre for Green Chemical Science, School of Chemical Sciences, University of Auckland, Private Bag 92019, Auckland 1010, New Zealand

* Correspondence: s.lo@auckland.ac.nz

Supplementary Material

Table S1: HPLC method established by [1] for analysis of phenolic compounds in red wine.

Time (min)	Solvent A (%)	Solvent B (%)	Solvent C (%)
**0**	45	55	0
**10**	25	75	0
**20**	0	100	0
**30**	0	100	0
**50**	0	90	10
**70**	0	85	15
**80**	0	80	20
**95**	0	60	40
**105**	0	55	45
**115**	45	55	0
**125**	45	55	0

Table S2: Quantities of flavonoids extracted with each solvent system and their rank in extraction efficiency. Values are averages of quantities measured in triplicate.

Solvent System			(+)-Catechin		(-)-Epicatechin		Quercetin	
Acetone (%)	H_2O (%)	EtOH (%)	mg /kg of pomace	Rank (out of 28)	mg /kg of pomace	Rank (out of 28)	mg of rutin hydrate /kg of pomace	Rank (out of 28)
80	20	0	174.1 (± 17.1)	1	259.3 (± 38.1)	4	49.5 (± 1.2)	15
70	30	0	169. 6 (± 6.9)	4	244.7 (± 5.0)	6	48.8 (± 5.3)	17
70	20	10	160.1 (± 18.7)	9	232.7 (± 30.6)	11	45.3 (± 3.4)	21
60	40	0	166.6 (± 24.2)	5	263.1 (± 24.1)	3	53.6 (± 11.1)	12
60	30	10	148.4 (± 25.7)	16	218.8 (± 39.5)	16	49.0 (± 14.3)	16
60	20	20	154.9 (± 17.0)	13	233.8 (± 13.2)	10	39.6 (± 2.1)	23
50	50	0	156.2 (± 19.8)	11	240.1 (± 28.8)	8	54.4 (± 5.8)	10
50	40	10	157.0 (± 32.1)	10	231.1 (± 39.0)	12	66.5 (± 4.2)	4
50	30	20	165.9 (± 16.3)	7	250.9 (± 25.3)	5	72.3 (± 39.1)	2
50	20	30	152.5 (± 9.7)	14	240.7 (± 56.5)	7	52.4 (± 20.1)	13
40	60	0	144.3 (± 16.2)	19	218.6 (± 35.6)	17	51.1 (± 1.3)	14
40	50	10	169.9 (± 28.3)	3	227.1 (± 18.8)	14	87.0 (± 46.4)	1
40	40	20	173.3 (± 23.9)	2	269.6 (± 34.1)	1	55.0 (± 1.3)	9
40	30	30	164.7 (± 18.4)	8	240.1 (± 8.9)	9	58.9 (± 1.0)	7
40	20	40	155.5 (± 13.9)	12	224.7 (± 26.7)	15	46.0 (± 5.8)	20
30	70	0	139.7 (± 42.7)	20	194.3 (± 44.0)	22	26.3 (± 2.2)	27
30	60	10	131.1 (± 23.8)	24	190.3 (± 28.5)	23	48.4 (± 2.0)	18
30	50	20	166.1 (± 22.5)	6	265.1 (± 37.6)	2	54.2 (± 0.6)	11
30	40	30	151.4 (± 10.3)	15	230.7 (± 19.8)	13	63.1 (± 7.9)	6
30	30	40	146.5 (± 20.8)	17	211.6 (± 10.9)	19	67.7 (± 36.0)	3
30	20	50	139.6 (± 8.7)	21	216.7 (± 28.9)	18	33.2 (± 11.7)	24
20	80	0	114.1 (± 12.0)	27	148.6 (± 11.1)	28	8.9 (± 0.7)	28
20	70	10	135.4 (± 13.9)	23	179.0 (± 35.8)	27	27.3 (± 5.1)	26
20	60	20	123.5 (± 21.0)	26	182.6 (± 45.0)	25	43.7 (± 13.6)	22
20	50	30	145.3 (± 10.2)	18	209.4 (± 13.9)	20	55.4 (± 5.8)	8
20	40	40	137.4 (± 17.2)	22	208.8 (± 25.3)	21	65.8 (± 3.3)	5
20	30	50	129.2 (± 24.3)	25	185.3 (± 40.8)	24	46.2 (± 1.0)	19
20	20	60	112.3 (± 11.3)	28	182.1 (± 18.4)	26	32.7 (± 2.8)	25

Figure S1: IR spectra of a) non-derivatised extracted residue of Pinot noir pomace; b) residue derivatised with octanoyl chloride; c) residue derivatised with lauroyl chloride; and d) residue derivatised with palmitoyl chloride.

a)

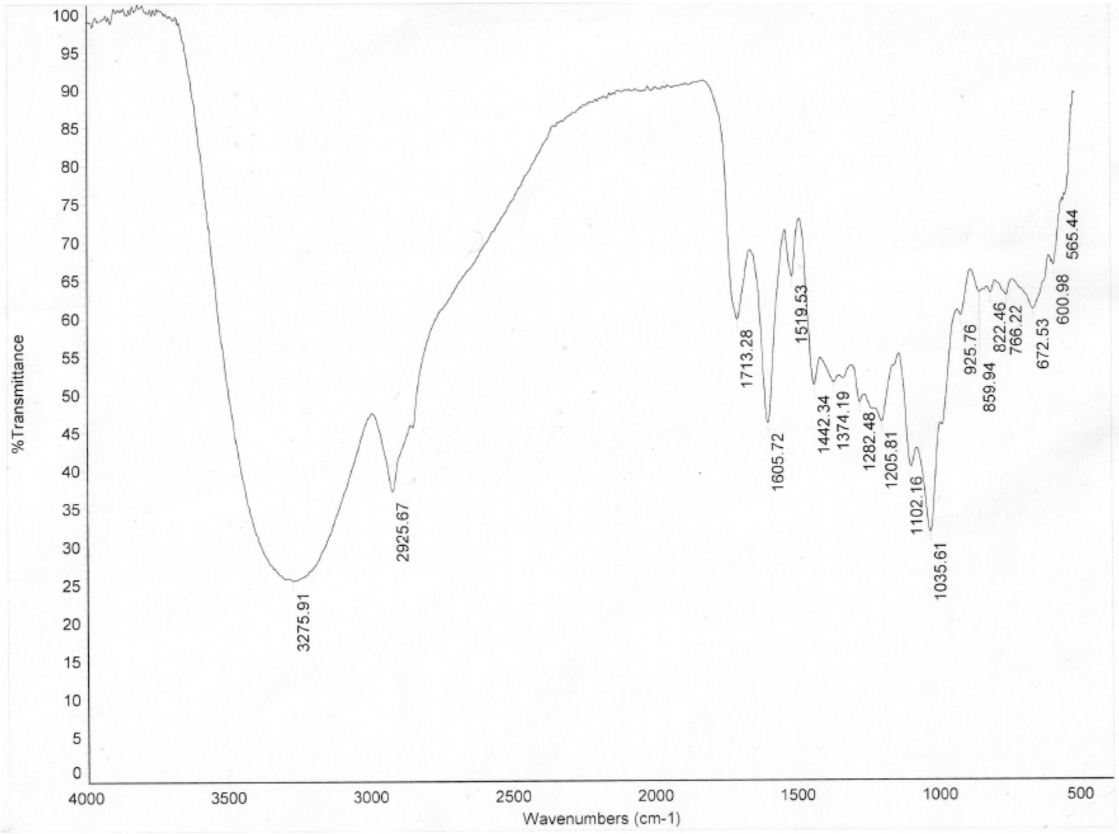

b)

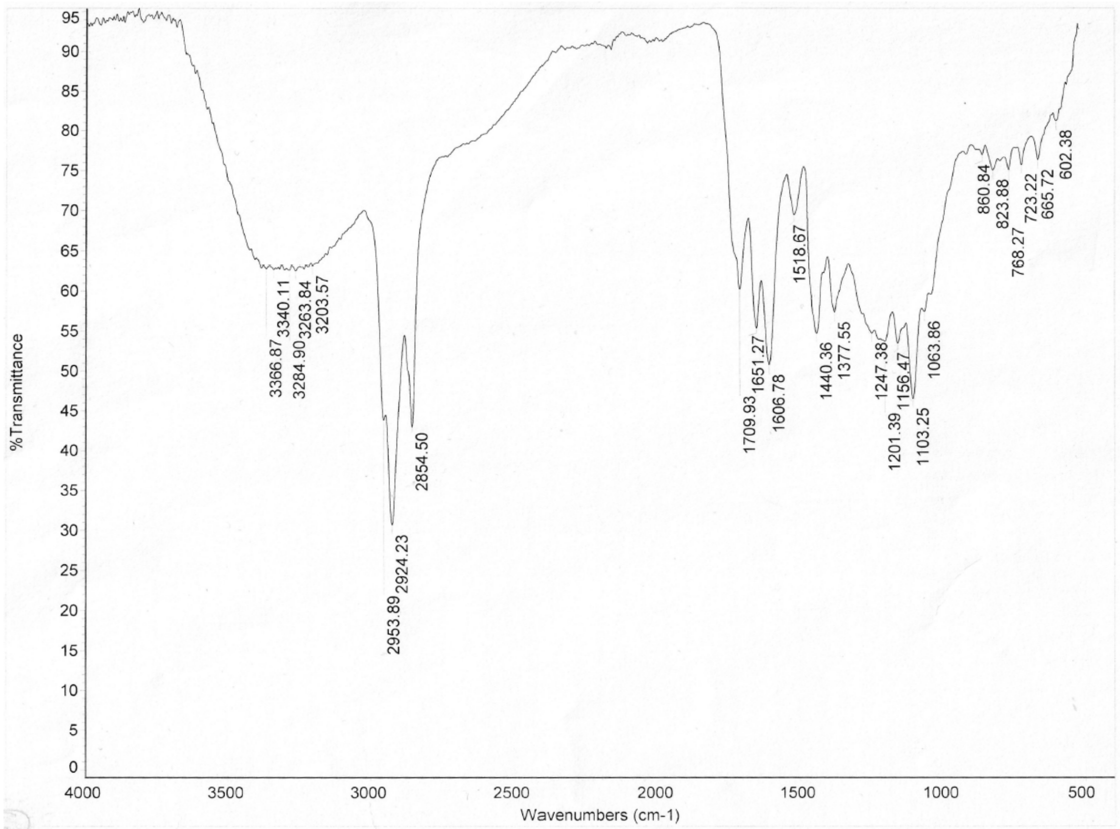
%Transmittance
Wavenumbers (cm-1)
3366.87
3340.11
3284.90
3263.84
3203.57
2953.89
2924.23
2854.50
1709.93
1651.27
1606.78
1518.67
1440.36
1377.55
1247.38
1201.39
1156.47
1103.25
1063.86
860.84
823.88
768.27
723.22
665.72
602.38

c)

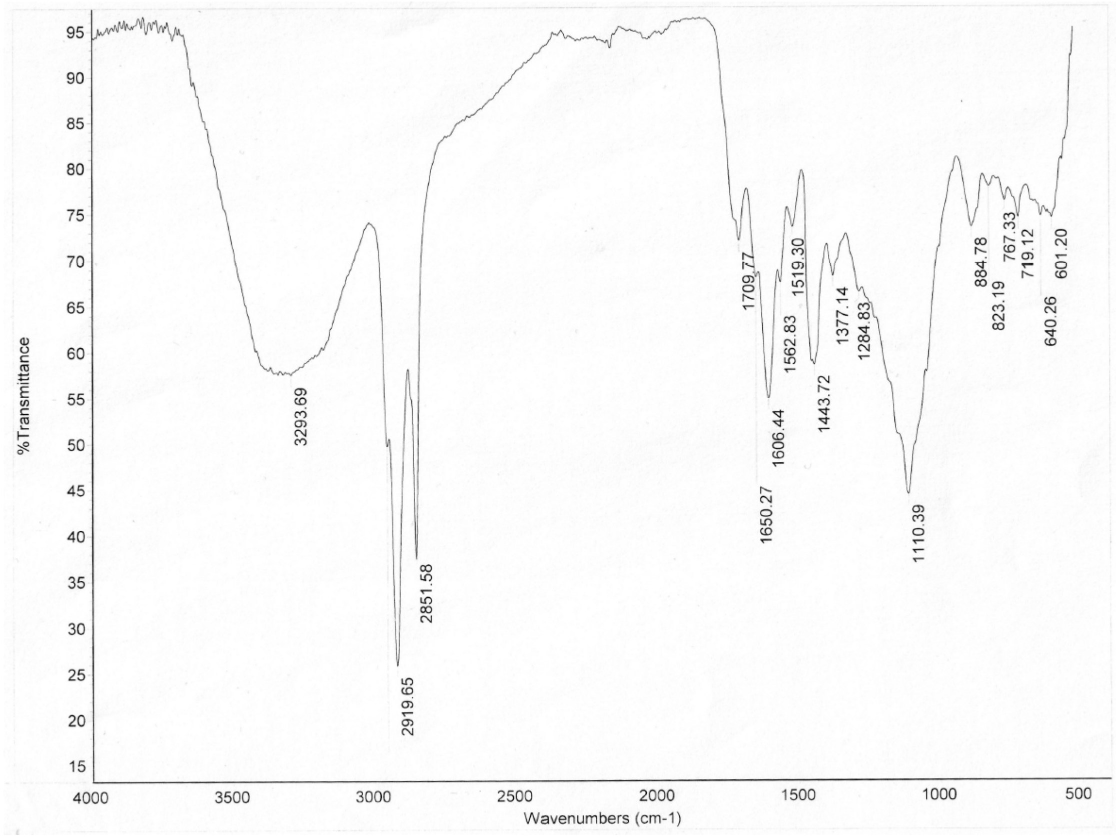
%Transmittance
Wavenumbers (cm-1)
3293.69
2919.65
2851.58
1709.77
1650.27
1606.44
1562.83
1519.30
1443.72
1377.14
1284.83
1110.39
884.78
823.19
767.33
719.12
640.26
601.20

d)

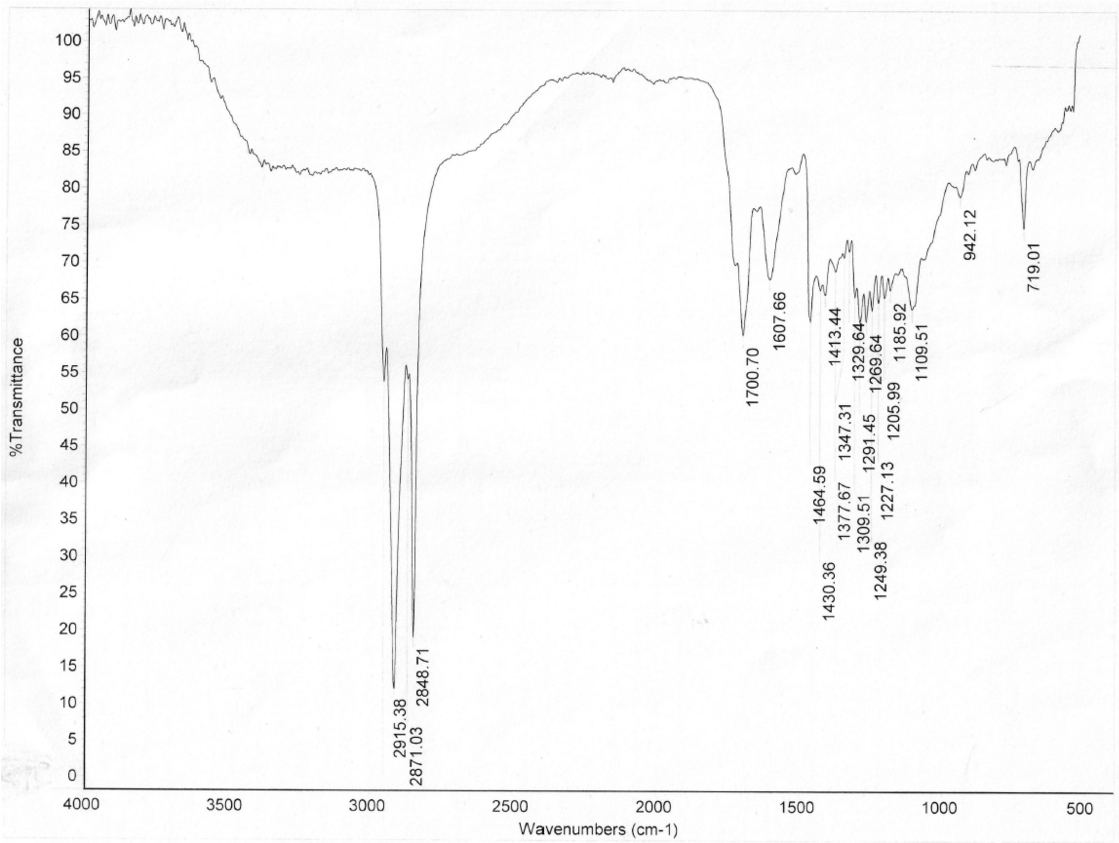
%Transmittance
Wavenumbers (cm-1)
2915.38
2871.03
2848.71
1700.70
1607.66
1464.59
1430.36
1413.44
1377.67
1347.31
1329.64
1309.51
1291.45
1269.64
1249.38
1227.13
1205.99
1185.92
1109.51
942.12
719.01

References

1. Kilmartin PA, Zou H, Waterhouse AL (2002) Correlation of Wine Phenolic Composition versus Cyclic Voltammetry Response. Am J Enol Vitic 53:294–302
